# ﻿*Alliumsunhangii* – a new species from section *Brevidentia* F.O.Khass. & Iengal. (Amaryllidaceae) from Southern Pamir-Alay, Uzbekistan

**DOI:** 10.3897/phytokeys.219.96464

**Published:** 2023-01-24

**Authors:** Furkat O. Khassanov, Sardorjon Pulatov, Temur Asatulloev, Ibrokhimjon Ergashov, Komiljon Sh. Tojibaev, Ziyoviddin Yusupov

**Affiliations:** 1 Institute of Botany, Academy of Sciences of Uzbekistan, Tashkent 100125, Uzbekistan Institute of Botany, Academy of Sciences of Uzbekistan Tashkent Uzbekistan; 2 CAS Key Laboratory for Plant Diversity and Biogeography of East Asia, Kunming Institute of Botany, Chinese Academy of Sciences, Kunming 650201, Yunnan, China Kunming Institute of Botany, Chinese Academy of Sciences Kunming China; 3 University of Chinese Academy of Sciences, Beijing, China University of Chinese Academy of Sciences Beijing China

**Keywords:** *Allium*, *Brevidentia*, Middle Asia, new taxon, phylogeny, taxonomy

## Abstract

A new species, *Alliumsunhangii***sp. nov.**, of the Middle Asiatic section Brevidentia F.O.Khass. & Iengal., (subgenusAllium, tribe Allioideae, Amaryllidaceae) is described. The species is a small plant from the Babatag Ridge in the Surkhandarya province of Uzbekistan. It is morphologically close to *Alliumbrevidens* Vved. in having initially dark violet filaments and three-cuspidate inner filaments, but differs by its small size and visibly unequal tepals as well as in the phylogenetic analysis based on ITS data.

## ﻿Introduction

*Allium*Linnaeus (1753), one of the largest genera in the Amaryllidaceae (Friesen et al. 2006; Li et al. 2010), has more than 1100 species worldwide (Govaerts et al. 2021). Members of the genus, such as garlic, leek, onion and shallot, are used as food, medicine and ornament (Herden et al. 2016) and are characterized by bulbs enclosed in a membranous, fibrous or reticulate tunic, free or basally connate tepals and often a subgynobasic style (Friesen et al. 2006). *Allium* has two probable diversity centers, one in South-Western and Middle Asia and in the Mediterranean region, and a smaller center is in western North America (Friesen et al. 2006; Nguyen et al. 2008). The most recent classification of *Allium*, by Friesen et al. (2006), based on molecular phylogenetic analyses, includes 15 subgenera and 72 sections.

Subgenus Allium, with more than 375 species and 35 subspecies, is the largest subgenus within *Allium*, and is one of three main evolutionary lines within the genus (Friesen et al. 2006; Fritsch and Friesen 2002). Subgenus Allium consists of two main groups (Hanelt 1992; Friesen et al. 2006); one has simple inner filaments while the other has three-cuspidate inner filaments. The newly described sections are supported by nuclear molecular data (Friesen et al. 2006) and have revealed the presence of centers of recent speciation in the Middle Asia, Pakistan, Iran, Afghanistan and the Middle East (Khassanov 2018). Also, results from whole chloroplast genome analyses are continuing and being compared with morphology to determine whether morphology-based taxonomy corresponds well to molecular data (Munavvarov et al. 2022)

Section Brevidentia F.O.Khass. & Iengal. was previously treated as a part of section Allium of subgenus Allium. Khassanov et al. (1997) divided section Allium into six sections (*Allium* s. str., *Crystallina* F.O.Khass. & Iengal., *Filidentia* F.O.Khass. & Iengal., *Brevidentia* F.O.Khass. & Iengal., *Spathulata* F.O.Khass. & R.M.Fritsch and *Multicaulea* F.O.Khass. & Iengal.). According to the last revised and updated classification of subgenus Allium (Khassanov 2018), section Brevidentia includes 12 species, most of which are in Middle Asia and adjacent areas. The main characteristics are purple filaments, the inner ones three-cuspidate, as well as a rounded purplish ovary with pocket-like mounds of the nectary tubes. Most species show S-to U-type, U-type anticlinal walls and (globular) convex periclinal walls of seeds (Yusupov et al. 2022).

In 2021, during grid mapping of the flora of the Surkhandarya province (Babatag Ridge, Zarkasa peak in Uzbekistan), we collected an interesting species of *Allium*. Comparisons of molecular and morphological characteristics showed it as a member of sect. Brevidentia. Morphologically, it resembles *A.brevidens* in its purple, three-cuspidate inner filaments, but differs in unequal tepals, which showed that it was a previously unknown characteristic for the species of *Allium*. Here, we propose it as new species and provide a comprehensive description based on morphological and molecular approaches.

## ﻿Materials and methods

### ﻿Plant material

A total of 14 specimens were collected in the summer of 2021. Material from the new species was collected in the Zarkasa (Babatag Ridge) peak, Surkhandarya province, Uzbekistan.

### ﻿DNA extraction, PCR amplification and sequencing

Leaves for molecular analysis were dried in silica gel upon collecting. Total DNA was isolated by the CTAB protocol (Doyle and Doyle 1987) from 1 g of well-dried leaves. ITS1 and ITS4 primers were from White et al. (1990). Polymerase chain reaction (PCR) was performed under the following conditions: 5 min of initial denaturation at 94 °C, 35 cycles of denaturation for 45 secs at 94 °C, annealing for 45 secs at 55 °C, and extension for 1–1.5 min at 72 °C, then a final extension at 72 °C for 5 min. PCR products were visualized using electrophoresis on 1.5% agarose TAE gel and sent to Beijing Genomics Institute (Shenzhen, China) for sequencing.

### ﻿Phylogenetic analyses

To assemble and edit complementary strands, we used Sequencher 4.1.4 software (Burland 2000). Clustal X (Jeanmougin et al. 1998) was used to align DNA sequences, which were then manually adjusted using MEGA 7.0 (Kumar et al. 2016). Analysis of parsimony was conducted in PAUP* 4.0b10 (Swofford and Sullivan 2003) using heuristic searches with TBR and 1000 random addition sequence replicates. Bootstrap support (BS) was estimated with 1000 replicates, each with 100 random addition sequence searches according to Felsenstein (1985). The major consensus trees constructed from a maximum of 1000 trees were saved. RAxML v 8.2.8 (Stamatakis 2014). The best-fitting nucleotide substitution model GTR + G model was determined for each dataset and 1000 bootstrap replicates were used for performing Maximum Likelihood (ML) analyses. Based on the Akaike information criterion (AIC) implemented in jModelTest2 on XSEDE (www.phylo.org). For Bayesian inference (BI) analyses, MrBayes version 3.1.2 (Huelsenbeck and Ronquist 2001) was utilized, with 10,000,000 generations with random trees sampled every 1000 generations. In the latter analysis, after discarding the first 25% of trees as burn-in, and in order to estimate posterior probabilities (PP) we constructed a 50% majority-rule consensus tree from the remaining trees.

A total of 28 ITS sequences were downloaded from NCBI and used for phylogenetic reconstruction. In order to confirm the systematic position of the new species we selected 8 sections of subgen. Allium and two species from subgen. Rhizirideum (see Appendix 1). The classification system in this study follows the nuclear-based molecular phylogenetic classification of Friesen et al. (2006).

## ﻿Results

### ﻿Taxonomic treatment

#### ﻿ Allium sunhangii

Taxon classificationPlantaeAsparagalesAmaryllidaceae

F.O.Khass., Tojibaev & Yusupov sp. nov.

25719019-0FCA-5D47-8DD7-28A08A8C6F00

urn:lsid:ipni.org:names:77311234-1

Figs 1 , 2 , 3

##### Type.

Uzbekistan. Surkhandarya province, Babatag Ridge, Zarkasa peak, 37.986537, 68.166650, 2251 m a.s.l., 22 June 2021, *S.O. Pulatov and O.A.Turdiboev 22062021001*. (TASH109001!, holotype; TASH109002! and TASH111001!, isotypes).

##### Description.

Bulbs 0.4–0.8 cm wide, 0.7–0.9 cm long, ovoid, solitary tunics reticulate, light brown, bulblets several, smooth, brownish. Scape terete, erect, 4.5–10 cm high, 1.0–1.2 mm wide. Spathe bivalved, persistent, ca 4 mm long, with short beak. Leaves 2–4, narrowly linear, longer than inflorescence, 6–12 cm long, 1.0–1.5 mm wide, semi-terete. Inflorescence lax, umbellate, hemispheric, 10 to 15-flowered. Flowers widely cup-shaped, nearly star-like, ca 5 mm long. Pedicels 2–3 times longer than tepals, at base with bracts. Tepals lanceolate-ovate, smooth, whitish with a dirty greenish-purple midvein, 2.5–4 mm long, outer tepals slightly longer than inner ones. Filaments 1.5–2.0 times longer than tepals, inner ones 3-cuspidate, filament bearing cusp 2 times longer than basal teeth. Style exerted from flowers. Capsule 2 mm in diam.

##### Diagnosis.

**Figure 1.**
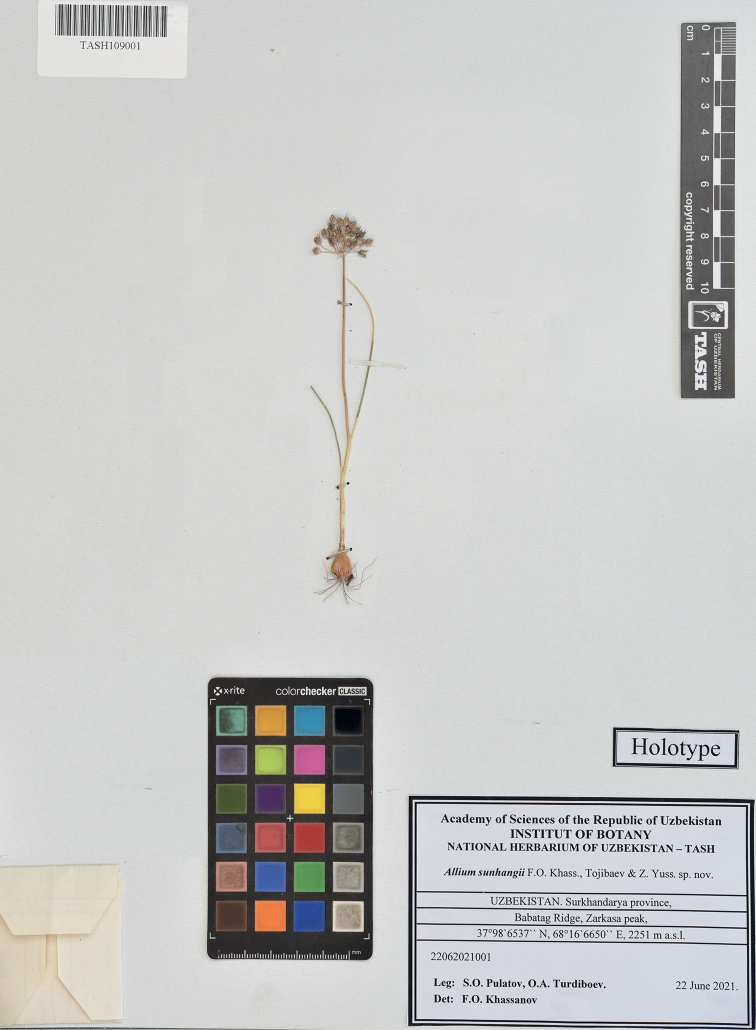
Holotype of *Alliumsunhangii* F.O.Khass., Tojibaev & Z.Yuss., sp. nov.

**Figure 2.**
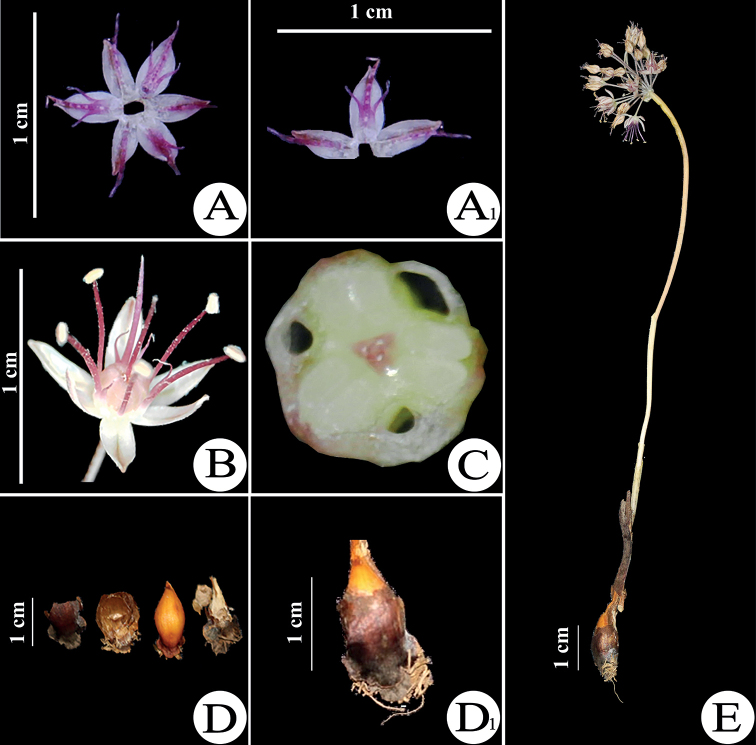
*Alliumsunhangii***A–A_1_** whole and longitudinal section of flower with teeth **B** view of single flower **C** cross section of pistil **D–D_1_** bulb tunic and bulb **E** general view of species without leaves.

This species is most similar to *Alliumbrevidens* Vved. (Fig. 1), from which it differs in a more compact habit, remaining small spathe with a short beak, unequal tepals and strongly exserted, dark violet filaments (Fig. 2).

##### Distribution and habitat.

**Figure 3.**
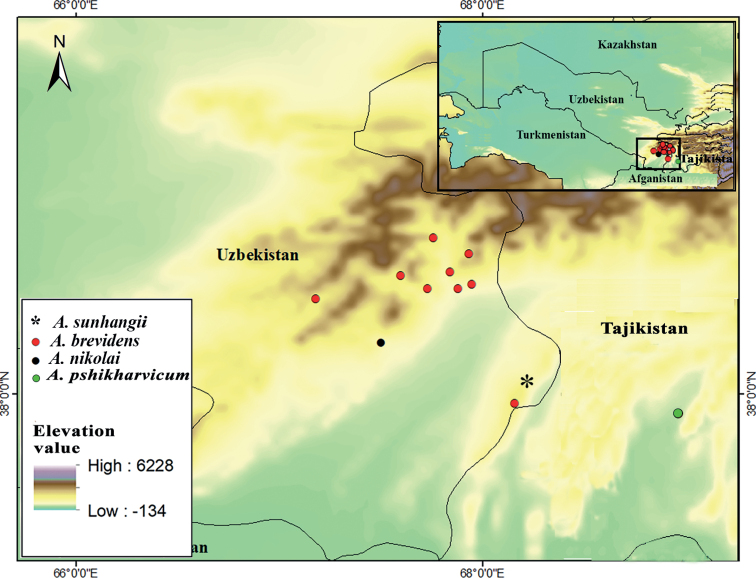
Distribution of *Alliumsunhangii*, *A.brevidens*, *A.nikolai* and *A.pshikharvicum*.

**Figure 4.**
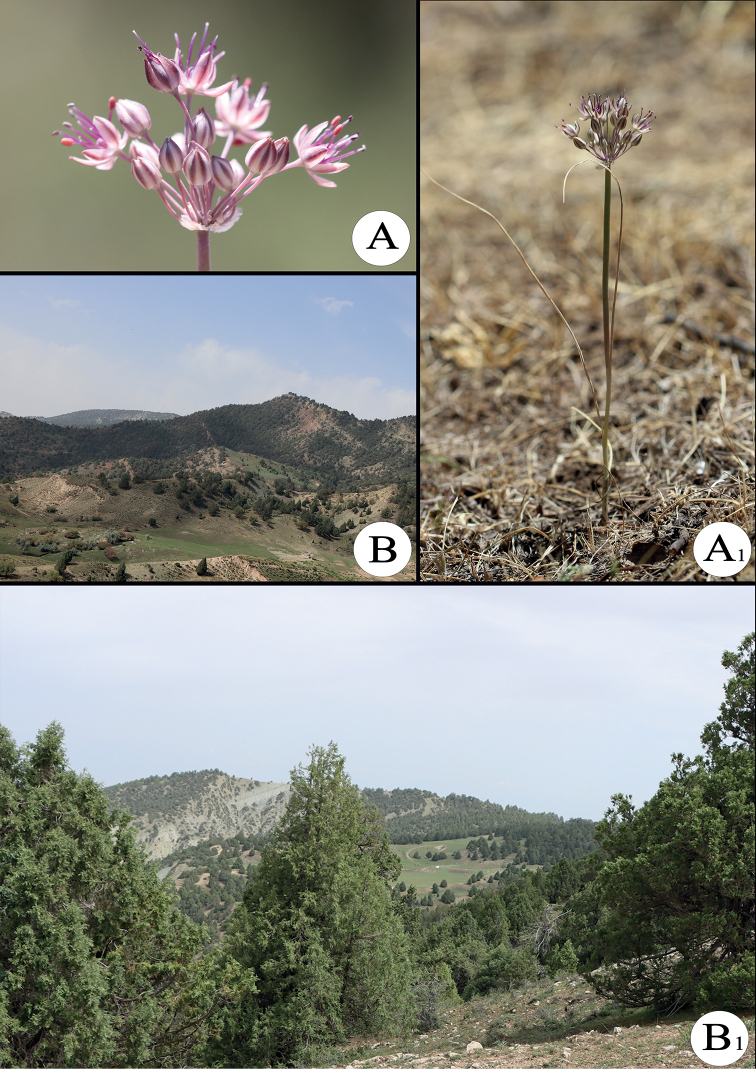
**A_1_–A_2_** inflorescence and general view of growing *Alliumsunhangii***B_1_–B_2_** Zarkasa peak and habitat landscape.

*Alliumsunhangii* is known from one population occurring to the south in the northwestern part of the Zarkasa peak, at 2251 m a.s.l. (Figs 3, 4). New species grows in continental and drier *Juniperus* forests (Fig. 4B_1_– B_2_) (*Juniperusseravschanica* Kom.) primarily on loamy soil, with shrubs (*Cotoneasternummularius* Fisch. & C.A.Mey., *Loniceranummulariifolia* Jaub. & Spach, *Rosacanina* L., *Rosaecae* Aitch.), perennial (*Convolvuluslineatus* L., *Dianthustetralepis* Nevski & Schischk., Eremurus *olgae* Regel, *Gentianaolivieri* Griseb., *Hypericumscabrum* L., *Malvaneglecta* Wallr., *Phlomisolgae* Regel, *Primulabaldshuanica* B. Fedtsch., *Ziziphorapamiroalaica* Juz.), annual and biennial (*Cousiniacandicans* Juz., *C.microcarpa* Boiss., *Daucuscarota* L., *Lactucaserriola* L., *Lappulamicrocarpa* (Ledeb.) Giirke, *Veronicacardiocarpa* (Kar. & Kir.) Walp.,) herbs and is always with dominance by *Carexpachystylis* J.Gay.

##### Etymology.

*Alliumsunhangii* is named after Prof. Sun Hang, one of the leading botanists at the Kunming Institute of Botany, Chinese Academy of Sciences, China, who actively promotes several projects within Central Asia.

##### Phenology.

*Alliumsunhangii* was flowering (Fig. 4 A_1_–A_2_) on 22 June, 2021 when we found its fruits began to mature at the same time. It is supposed that flowering starts in about late May and/or early June. As we visited this area only once, we are not sure when fruiting finishes.

##### Conservation status.

*Alliumsunhangii* is so far only known from two closely spaced localities. The total distribution area of this species is around 5 km^2^. The total number of individuals does not exceed 41. However, the new species is categorized as ‘Data Deficient’ (DD) according to IUCN (2019) criteria.

### ﻿Phylogenetic analysis

**Figure 5.**
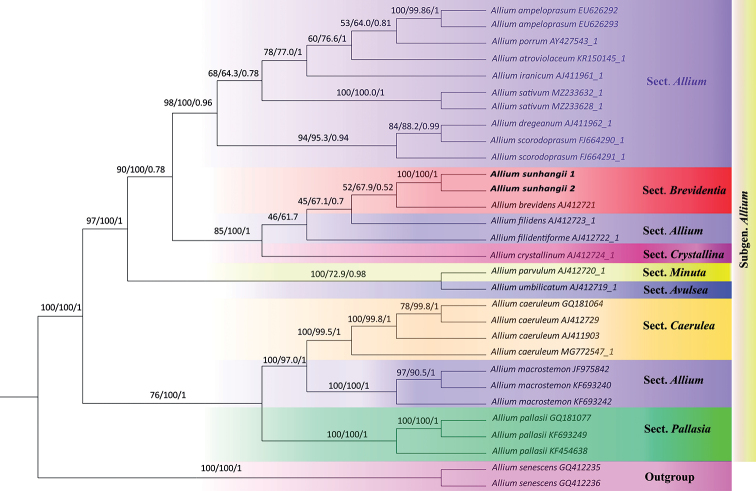
Phylogenetic tree inferred from MP, ML and BI (bootstrap support and posterior probabilities are given on branches, respectively), showing location of the *Alliumsunhangii*.

*Alliumsunhangii* was placed in the section Brevidentia (subgen.Allium) in all phylogenetic analyses (MP, ML and BI) (Fig. 5). Phylogenetic tree based on ITS data suggests that the new species closely related to *A.brevidens*.

## ﻿Discussion

*Alliumsunhangii* is morphologically close to *A.brevidens* in having initially dark violet filaments. However, it differs in a more compact habit, remaining small spathe with a short beak, unequal tepals and strongly exserted, dark violet filaments. Compared to all known species of Alliumsect.Brevidentia, the new species differs by having leaves longer than scape, spathe with rather small beak ca. 3 mm long; tepals whitish with greenish midvein. Most significantly, the new species has lax (vs dense) and umbellate (vs globose) inflorescence, and also fewer flowers, 10–25 (vs 30–50) (Table 1). In phylogenetic tree the new species and *A.brevidens* were placed along with the species of sect. Allium. Similarly, according to some unsolved reasons, species of the section Allium were placed in different positions in the previous studies (Friesen et al. 2006; Li et al. 2010). Accordingly, our phylogenetic analysis was also consistent with those phylogenetic analyses. However, *A.sunhangii* can be distinguished morphologically and geographically from the representatives of sect. Allium. In consistence of morphologic evidence, the position of *A.sunhangii* and *A.brevidens* in the phylogenetic tree supports that they are most relative and the new species belongs to sect. Brevidentia. Also, the distribution of the new species and the related species may also slightly support this arrangement. Thus, current molecular and morphological data support the recognition of *A.sunhangii* as a new species of Alliumsect.Brevidentia.

**Table 1.** Comparison in morphology between *Alliumsunhangii* sp. nov. and *A.brevidens*.

Characters	*A.sunhangii*	*A.brevidens*
Bulb	smooth	reticulate
Scape	4.5–10 cm	20–30 cm
Leaf	longer than scape	shorter than scape
Pedicels	2–3 times longer than tepals	3–8 times longer than tepals
Spathe	remaining	falling
Tepals	unequal (inners – 2 mm lg., outers – 3 mm lg.)	equal (inners and outers – 3.5–4.0 mm lg.)
Filaments	1.5–2.0 times longer than tepals	slightly longer than tepals

### ﻿Key for determination of species belonging to sect. Brevidentia

**Table:** 

1	Inner filaments simple, triangular-subulate	***A.miserabile***
–	Inner filaments 3 (or 5) cuspidate, the lateral sterile cusps shorter than the median anther- bearing cusp	**2**
2	Outer filaments with two obtuse teeth at base	***A.hedgei***
–	Outer filaments simple	**3**
3	Leaves normally twisted	**4**
–	Leaves normally straight	**6**
4	Perianth (6)7 mm long	***A.ophiophyllum***
–	Perianth 3.0–4.5 mm long	**5**
5	Perianth lilac with purple midvein; filaments violet, twice as long as tepals	***A.circumflexum***
–	Perianth lilac-greenish with green midvein; filaments whitish, shorter than tepals	***A.michaelis***
6	Filaments ciliate at the base, bracteoles present	**7**
–	Filaments glabrous, bracteoles absent	**11**
7	Bulblets with subcrystalline tunic	***A.brevidentiforme***
–	Bulblets without subcrystalline tunic	**8**
8	Plants to 60–80 cm tall; inflorescence dense, globose, flowers 30–50	**9**
–	Plants 10–30 cm tall; inflorescence lax, umbellate, flowers 10–25	**10**
9	Scape ca 80 cm tall; inflorescence dense, tepals greenish red with green midvein,	***A.pshikharvicum***
–	Scape ca 30 cm tall; inflorescene loose, tepals white with purple midvein	***A.brevidens***
10	Leaves shorter than scape; spathe with beak to 1 cm long; tepals rose colored, with purple midvein	***A.nikolai***
–	Leaves longer than scape; spathe with beak ca 3 mm long; tepals whitish with greenish midvein	***A.sunhangii***
11	Outer tunic reticulate-fibrous; perianth urceolate-campanulate, whitish	***A.ionandrum***
–	Outer tunic coriaceous; perianth, widely bell-shaped, purple or viole	***A.micranthum***

### ﻿Members of Alliumsect.Brevidentia

Sect. ***Brevidentia*** F.O.Khass. & Yengal. in Ozturk, Sećmen & Gork (Eds.) Plant Life in South West Asia. Ege Univ. Press, Izmir:147 (1996).

**Figure 6.**
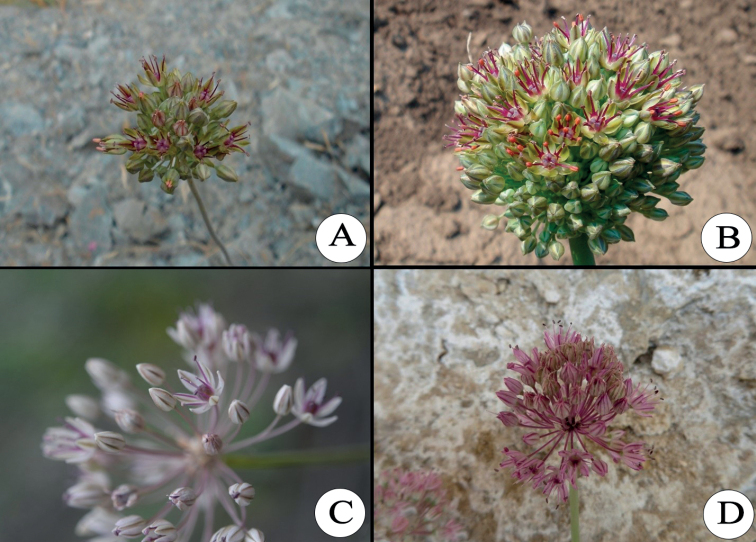
The species of section Brevidentia**A***A.brevidens***B***A.pshikharvicum***C***A.michaelis***D***A.nikolai*.

1. ***A.brevidens*** Vved. in Bot. Mater. Gerb. Glavn. Bot. Sada R.S.F.S.R. 5: 89 (1924). Holotype: Uzbekistan. Bukhara Khanate, Hissar distr., hills on the southern slopes of Hissar range, near Karatag, (in Russian). 20 May 1913, *A.I. Michelson 1721* (lectotype LE; designated by Khassanov in Flora of Uzbekistan 1: 61 (2017)). Distribution: Middle Asia (Southern Pamir-Alai): Tajikistan, Uzbekistan (Fig. 6A).
2. ***A.brevidentiforme*** Vved. in Opred. Rast. Sred. Azii 2: 315, 78 (1971). Holotype: Uzbekistan. Kashkadarja valley, Igri-su river, right bank, Juniper forests (in Russian), 6 July 1955, fl., *Pjataeva*, *Tsukerwanik 1617* (TASH000341!). Distribution: Western Pamir Alay (Hissar Range): Uzbekistan.
3. ***A.circumflexum*** Wendelbo in Acta Horti Gothob. 28: 22 (1966). Type: Iran. Prov. Bamian, Band-e-Amir, rich limestone steppe vegetation, 2900 m, 29 June 1962, leg. *Hedge & Wendelbo 4803* (holotype BG, isotypes E; TASH000348!). Distribution: Afghanistan.
4. ***A.hedgei*** Wendelbo in Acta Horti Gothob. 28: 20 (1966). Type: Afghanistan. Prov. Mazar-i-Sharif, Takht-i-Rustam, near Samangan (Aybak), dry slopes, 1200 m, 10 June 1962, leg. *Hedge & Wendelbo 3990* (holotype BG, isotypes E; TASH000390!). Distribution: Afghanistan.
5. ***A.ionandrum*** Wendelbo in Bot. Not. 121: 270 (1968). Type: Afghanistan, Urgun. 35 km NW Urgun, 32°27'N, 69°07'E, versus Surmat, 33°27'N, 69°02'E, 2200–2400 m. 10 June 1967, Per Wendelbo *35915* (holotype W, isotype B, MUN). Distribution: Afghanistan.
6. ***A.michaelis*** F.O.Khass. & Tojibaev in Linzer Biol. Beitr. 41(2): 1059 (2009). Holotype: Uzbekistan. Western Tian-Shan, Kurama Range, near Ujgursaj village, 40°54'54"N, 71°03'27"E, 563 m, 24 May 2009, *Khassanov*, *Tojibaev*, *Keusgen* (TASH000424!). Distribution: Ferghana valley (Uzbekistan, Kyrgyzstan) (Fig. 6C).
7. ***A.micranthum*** Wendelbo in Biol. Skr. 10, No. 3 (Symb. Afghan. 4): 178 (1959) (as cited in Nasir 1975, 22. p). Type: Afghanistan. Kurram valley, Afghanistan, December 1879, *Dr. J.R.T. Aitchison 228*, (holotype K). Distribution: Afghanistan.
8. ***A.miserabile*** Wendelbo in Nytt Mag. Bot., Oslo xiv. 104 (1967). Type: Pakistan. Flora of West Pakistan, Kohat, Kohat to Thal, c. 20 km from Kohat, Rocky slope on a small hillock, c. 675 m. 26 May 1965, *Jennifer Lammond 1549* (holotype E). Distribution: Afghanistan.
9. ***A.nikolai*** F.O.Khass. & Achilova in Opred. Rast. Sred. Azii 11: 497 (2015). Neotype: Uzbekistan. 25 km eastern Bajssun town, gypsaceous slopes under the shrubs, 23 July 2013, *Yusupov et al. s. n.* (TASH). Distribution: Uzbekistan (Kelif-Sherabad mountain range). Uzbekistan (Fig. 6D).
10. ***A.ophiophyllum*** Vved. in Trudy Sredne-Aziatsk. Gosud. Univ., Ser. 8b, Bot. 3: 8 (1928) (as cited in Khassanov and Yusupov 2022, 415. p). Type: Uzbekistan. Montes Meridionales: Sogdiano-transoxanae. Ad declivia argilloso-arenosa gypsacea, elevationis Chaudak-tau haud procul a pago Dzharkurgan, 30 April 1928, *Vvedensky s. n.* (TASH000440!, isotype K, W, MBG, LE, MW). Distribution: Southern Pamir-Alay (Uzbekistan, Tajikistan).
11. ***A.pshikharvicum*** (R.M. Fritsch & F.O.Khass.) F.O. Khass & Z.Yuss. in M.Ozturk et al. Biodiversity, Conservation and Sustainability in Asia. Volume 2: Prospects and Challenges in South and Middle Asia. Springer, 2022, p. 415. Type: Tajikistan, Darvaz Range, the road from pass Khoburabot between Robot and soldier post, steep stony-loamy slopes, in SE to SW exposition; 2200 m, 38°33'17"N, 70°48'07"E, leg. *Fritsch*, *Keusgen*, *Hissoriev*, *Kudratov 6199*, (Holotype GAT!, isotypes GAT!, TAD!). Distribution: Southern Pamir Alay (Darwaz Range): Tajikistan (Fig. 6B).
12. ***A.sunhangii*** F.O.Khass., Tojibaev & Yusupov sp. nov. Holotype: Uzbekistan. Surkhandarya province, Babatag Ridge, Zarkasa peak, 37.986537, 68.166650, 2251 m, 22 June 2021, *S.O. Pulatov and O.A. Turdiboev 22062021001* (TASH109001!, Holotype). Distribution: Middle Asia: Southern Pamir-Alay (Babatag ridge). Uzbekistan.

## Supplementary Material

XML Treatment for Allium sunhangii

## ﻿Appendix 1

**Table A1.** List of the GenBank accession numbers of the ITS sequences of sampled species in this study. Sequences generated in this study are marked with asterisks (*).

Species	Location, collector, herbarium voucher /Reference	GenBank Accession Number
*Alliumampeloprasum* L.	Spain, collected by C. M. Messiaen,BF-ALL-015/Hirschegger et al. 2009	EU626292
*Alliumampeloprasum* L.	Argentina, BF-ALL-001/ Hirschegger et al. 2009	EU626293
*Alliumatroviolaceum* Boiss.	Iran: N khorasan. collected by Yousef Saeedi/ Ghorbani et al. 2015 (unpubl. res.)	KR150145.1
*Alliumdregeanum* Kunth	South Africa, Tax 5772/Friesen et al. 2016	AJ411962.1
*Alliumiranicum* (Wendelbo) Wendelbo	Iran:Elburz range, Karaj valley, Asara, Tax 3969/Friesen et al. 2016	AJ411961
*Alliummacrostemon* Bunge	China: Shanxi, collected by Li Qinqin, He Xingjin, Hexj0473/Li et al. 2011	JF975842
*Alliummacrostemon* Bunge	China: Yunnan, Hutiaoxia, collected by D.Q Huang, H11100509/He X and Huang D 2013 (unpubl. res.)	KF693240
*Alliummacrostemon* Bunge	China: Yunnan, Kunming, collected by Q.Q Li, L20081102/He and Huang 2013 (unpubl. res.)	KF693242
*Alliumporrum* L.	NVRS 01 4549/Ricroch et al. 2005	AY427543.1
*Alliumsativum* L.	Iran: Hamadan/Fakhrfeshani et al. 2021 (unpubl. res.)	MZ233628.1
*Alliumsativum* L.	Iran: Hamadan/Fakhrfeshani et al. 2021 (unpubl. res.)	MZ233632.1
*Alliumscorodoprasum* L.	Slovenia, collected by P. Hirschegger, BF-ALL-042/Hirschegger et al. 2009	FJ664290.1
*Alliumscorodoprasum* L.	Slovenia, collected by P. Hirschegger, BF-ALL-044/Hirschegger et al. 2009	FJ664291.1
*Alliumumbilicatum* Boiss.	Iran:Teheran, Tax 2646/Friesen et al. 2001 (unpubl. res.)	AJ412719.1
*Alliumbrevidens* Vved.	Uzbekistan:Hissar Mts, Tax 5037/Friesen et al. 2001 (unpubl. res.)	AJ412721
*Alliumcaeruleum* Pall.	Russia:B. G. Moscow, Tax 1525/Friesen et al. 2001 (unpubl. res.)	AJ411903
*Alliumcaeruleum* Pall.	Kazakhstan:Chu-Ili Mts., Tax 3735/Friesen et al. 2001 (unpubl. res.)	AJ412729
*Alliumcaeruleum* Pall.	Collected by He XJ & Zhang XL, 97609/Li et al. 2010	GQ181064
*Alliumcaeruleum* Pall.	Kazakhstan, ipbb_2.1.12.1/Turuspekov et al. 2018 (unpubl. res.)	MG772547.1
*Alliumfilidens* Regel	Kazakhstan:Karatau Mts., Tax 3674/Friesen et al. 2001 (unpubl. res.)	AJ412723.1
*Alliumfilidentiforme* Vved.	Tajikistan:Schakhristan Pass, Tax 2573/Friesen et al. 2001 (unpubl. res.)	AJ412722.1
*Alliumcrystallinum* Vved.	Uzbekistan:Chakchar Mts., Tax 3662/Friesen et al. 2001 (unpubl. res.)	AJ412724.1
*Alliumparvulum* Vved.	Kyrgyzstan:Tallas Mts., Tax 5055/Friesen et al. 2001 (unpubl. res.)	AJ412720.1
*Alliumpallasii* Murray	Collected by He XJ & Zhang XL, 97603/Li et al. 2010	GQ181077
*Alliumpallasii* Murray	654026120714039/Li and Fan 2013 (unpubl. res.)	KF454638
*Alliumpallasii* Murray	654026120714039/He and Huang 2013 (unpubl. res.)	KF693249
*Alliumsenescens* L.	Collected by H.J.Choi, 070001 (KH)/Jang et al. 2009 (unpubl. res.)	GQ412235
*Alliumsenescens* L.	Collected by H.J.Choi et al., 010009 (CBU)/Jang et al. 2009 (unpubl. res.)	GQ412236
***Alliumsunhangii* F.O.Khass., Tojibaev & Yusupov sp. nov.**	Uzbekistan, Babatag Ridge: Zarkasa peak, collected by S.O. Pulatov and O. Turdiboev, 22062021001	**OP642456***
***Alliumsunhangii* F.O.Khass., Tojibaev & Yusupov sp. nov.**	Uzbekistan, Babatag Ridge: Zarkasa peak, collected by S.O. Pulatov and O. Turdiboev, 22062021002	**OP642457***

## References

[B1] Burland TG (2000) DNASTAR’s Lasergene Sequence Analysis Software. In: Misener S, Krawetz SA (Eds) Bioinformatics Methods and Protocols. Methods in Molecular Biology (Vol. 132). Humana Press, Totowa. 10.1385/1-59259-192-2:71 10547832

[B2] Doyle JJ Doyle JL (1987) A Rapid DNA Isolation Procedure for Small Quantities of Fresh Leaf Tissue. Phytochemical Bulletin 19: 11–15.

[B3] Felsenstein J (1985) Phylogenies and the comparative method. The American Naturalist 125: 1–15. 10.1086/284325 31094602

[B4] Friesen N Fritsch R Blattner F (2006) Phylogeny and new intrageneric classification of *Allium* (Alliaceae) based on nuclear ribosomal DNA ITS sequences. Aliso: A Journal of Systematic and Floristic Botany 22: 372–395. 10.5642/aliso.20062201.31

[B5] Fritsch R Friesen N (2002) Evolution, domestication and taxonomy In: Currah L (Ed.) *Allium* crop science: recent advances, UKCABI Publishing, Wallingford, 5c30. 10.1079/9780851995106.0005

[B6] Govaerts R Kington S Friesen N Fritsch R Snijman D Marcucci R Silverstone-Sopkin P Brullo S (2021) World checklist of Amaryllidaceae. Facilitated by the Royal Botanic Gardens, Kew. https://wcsp.science.kew.org/

[B7] Hanelt P (1992) Ovule number and seed weight in the genus *Allium* L. Institute of Plant Genetics and Crop Plant Research. https://worldveg.tind.io/record/19730/

[B8] Herden T Hanelt P Friesen N (2016) Phylogeny of AlliumL.subgenusAnguinum (G. Don. ex W.D.J Koch) N. Friesen (Amaryllidaceae). Molecular Phylogenetics and Evolution 95: 79–93. 10.1016/j.ympev.2015.11.004 26639102

[B9] Huelsenbeck JP Ronquist F (2001) MRBAYES: Bayesian inference of phylogenetic trees. Bioinformatics 17(8): 754–755. 10.1093/bioinformatics/17.8.754 11524383

[B10] IUCN [International Union for Conservation of Nature] (2019) Guidelines for the IUCN Red List categories and criteria. Version 13. IUCN, Gland.

[B11] Jeanmougin F Thompson J Gouy M Higgins D Gibson T (1998) Multiple sequence alignment with Clustal X. Trends in Biochemical Sciences 23(10): 403–405. 10.1016/S0968-0004(98)01285-7 9810230

[B12] Khassanov FO (2018) Taxonomical and Ethnobotanical Aspects of *Allium* Species from Middle Asia with Particular Reference to Subgenus Allium. In: Shigyo M, Khar A, Abdelrahman M (Eds) The *Allium* Genomes. Compendium of Plant Genomes. Springer, Cham. 10.1007/978-3-319-95825-5_2

[B13] Khassanov FO Yusupov Z (2022) Prospects and Challenges in South and Middle Asia. In: Öztürk M Khan SM Altay V Efe R Egamberdieva D Khassanov F (Eds) Prospects and Challenges in South and Middle Asia.Springer Nature, Switzerland, 403–433. 10.1007/978-3-030-73943-0_22

[B14] Khassanov FO Öztürk M Secmen Ö Görk G (1997) Conspectus of the wild growing *Allium* species of Middle Asia. In: Öztürk MA (Ed.) Plant life in Southwest and Central Asia.Turkey Ege University Press, Izmir, 141–159.

[B15] Kumar S Stecher G Tamura K (2016) MEGA7: Molecular evolutionary genetics analysis version 7.0 for bigger datasets. Molecular Biology and Evolution 33(7): 1870–1874. 10.1093/molbev/msw054 27004904 PMC8210823

[B16] Li QQ Zhou SD He XJ Yu Y Zhang YC Wei XQ (2010) Phylogeny and biogeography of *Allium* (Amaryllidaceae: Allieae) based on nuclear ribosomal internal transcribed spacer and chloroplast rps16 sequences, focusing on the inclusion of species endemic to China. Annals of Botany 106(5): 709–733. 10.1093/aob/mcq177 20966186 PMC2958792

[B17] Linnaeus C (1753) Species Plantarum (Vol. 1). Impensis Laurentii Salvii, Stockholm, 557 pp.

[B18] Munavvarov A Yusupov Z Ergashov I Tojibaev KSH Deng T Sun H (2022) Complete chloroplast genomes of ten species from subgenusAllium (*Allium*, Amaryllidaceae). Plant Diversity of Central Asia 1(2): 67–81. 10.54981/PDCA/vol1_iss2/a3

[B19] Nasir E (1975) Alliaceae. In: Nasir E, Ali S (Eds) Flora of Pakistan. University of Karachi, Pakistan, 31 pp.

[B20] Nguyen NH Driscoll HE Specht CD (2008) A molecular phylogeny of the wild onions (*Allium*; Alliaceae) with a focus on the western North American center of diversity. Molecular Phylogenetics and Evolution 47(3): 1157–1172. 10.1016/j.ympev.2007.12.006 18226928

[B21] Stamatakis A (2014) RAxML version 8: A tool for phylogenetic analysis and post-analysis of large phylogenies. Bioinformatics 30(9): 1312–1313. 10.1093/bioinformatics/btu033 24451623 PMC3998144

[B22] Swofford DL Sullivan J (2003) Phylogeny inference based on parsimony and other methods using PAUP*. The phylogenetic handbook: a practical approach to DNA and protein phylogeny, 160–206.

[B23] White TJ Bruns T Lee S Taylor J (1990) Amplification and direct sequencing of fungal ribosomal RNA genes for phylogenetics. In: Innis MA Gelfand DH Sninsky JJ White TJ (Eds) PCR Protocols: a guide to methods and applications.Academic Press, San Diego, 315–322. 10.1016/B978-0-12-372180-8.50042-1

[B24] Yusupov Z Ergashov I Volis S Makhmudjanov D Dekhkonov D Khassanov F Tojibaev K Deng T Sun H (2022) Seed macro-and micromorphology in *Allium* (Amaryllidaceae) and its phylogenetic significance. Annals of Botany 129(7): 869–911. 10.1093/aob/mcac067 35696666 PMC9292631

